# Pulmonary Sarcoidosis following Etanercept Treatment

**DOI:** 10.1155/2012/724013

**Published:** 2012-12-23

**Authors:** Kuljeet Bhamra, Richard Stevens

**Affiliations:** Department of Rheumatology, Wycombe General Hospital, Queen Alexander Road, Wycome, Buckinghamshire HP11 2TT, UK

## Abstract

Tumour necrosis factor (TNF) is an important cytokine involved in the pathology of a number of inflammatory conditions, and thus blockade with anti-TNF therapies is becoming the cornerstone in managing such diseases. With increasing use, evidence is collected for the association of sarcoid-like granulomatous disease developing after the initiation of anti-TNF-**α** therapy, with disease reversal after discontinuation.

## 1. Introduction

We report a case of a 37-year-old married Pakistani-born woman, resident in the UK since the age of 19, who developed pulmonary sarcoidosis on treatment with etanercept for psoriatic arthritis. She first presented in 2009 with dactylitis affecting her left foot during the post partum period. She later went onto develop psoriatic plaques over her limbs, scalp, and trunk with worsening small and large joint arthritis. Her psoriatic arthritis was treated first with sulphasalazine. Due to side effects, her treatment was changed to methotrexate, with the later addition of leflunomide. Despite full-dose combination DMARD therapy and maintenance low-dose oral prednisone (5–10 mg/day), her disease remained active, requiring intramuscular and on occasion intravenous pulsed methyl-prednisolone to achieve even indifferent control and allow her to cope with looking after her 3 young children (PsARC score tender joints (TJ) 5, swollen joints (SJ) 6, physician global (PhG) 4/5, patient global (PtG) 4/5, DAS28 = 6.12, CRP 40 mg/L). Following screening, including a full history of potential exposure to tuberculosis (TB) and a normal baseline chest X-ray, she was commenced on etanercept 50 mg weekly sub.cut. in April 2010 and leflunomide was discontinued. She made a prompt response, achieving near joint and skin remission. (PsARC at three months TJ 0, SJ 2, PhG 1, PtG 2, CRP 1 mg/L, DAS28 = 2.03) and her maintenance prednisolone was phased out over the subsequent 3 months. She discontinued methotrexate on her own initiative 4 months after starting etanercept. She remained well until seven months into her etanercept treatment when she presented to clinic with a 3-week history of persistent dry cough and mild exertional breathlessness, having just returned from a 5-week stay in Pakistan.

**Figure 1:**
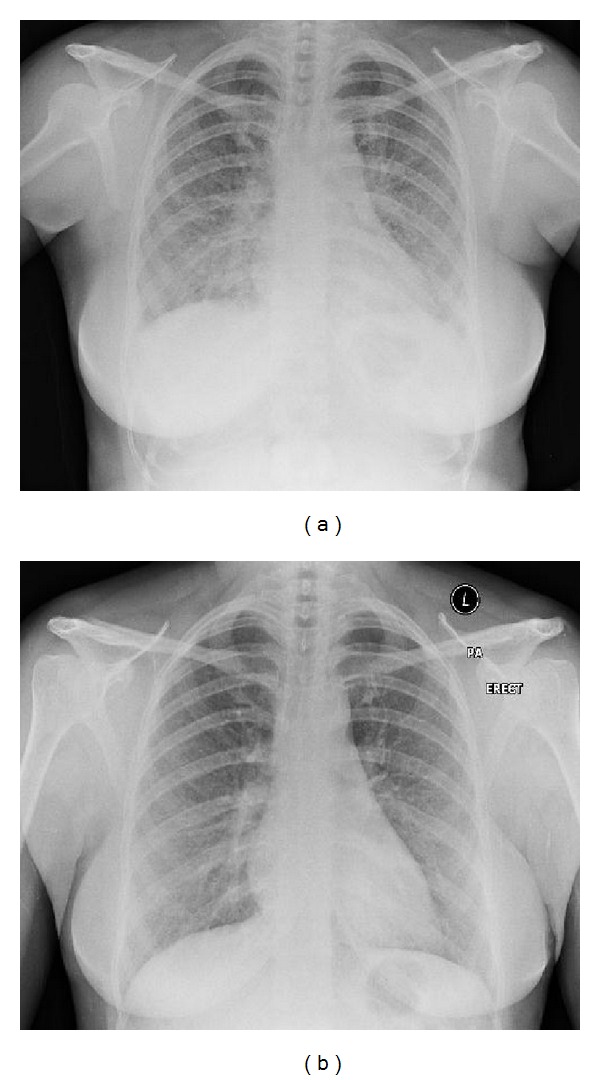
Chest X-ray pre-and postdiscontinuation of anti TNF-*α* therapy and commencement of oral prednisolone. (a) Chest X-ray with widespread reticulonodular shadowing with prominence of the of right hilum suggestive of lymphadenopathy pre-anti-TNF-*α* therapy. (b) Chest X-ray showing improvement in the extent of reticulonodular shadowing post-discontinuation of anti-TNF-*α* therapy and the commencement of oral prednisolone.

Chest X-ray showed widespread reticulonodular shadowing with prominence of the of right hilum suggestive of lymphadenopathy (Figure 1(a)); staining for acid fast bacilli and TB elispot both negative, serum ACE 49.9 U/L (20–70), corrected calcium 2.4 mmol/L (2.10–2.55).

**Figure 2:**
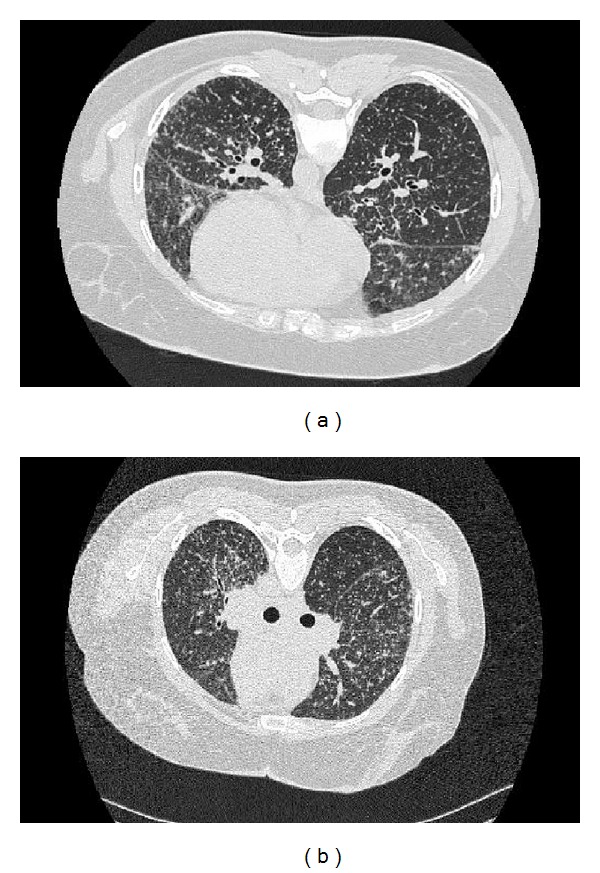
High resolution computed tomography showed multiple small nodules throughout the lung, few peribronchovascular nodules, with multiple enlarged mediastinal and hilar lymph nodes.

High resolution computed tomography showed multiple small nodules throughout the lung, few peribronchovascular nodules, with multiple enlarged mediastinal and hilar lymph nodes (Figures 2(a)-2(b)). Bronchoscopy performed revealed the presence of endobronchial nodes bilaterally; two transbronchial biopsies were taken from the right lower lobe with aid of screening. 

**Figure 3:**
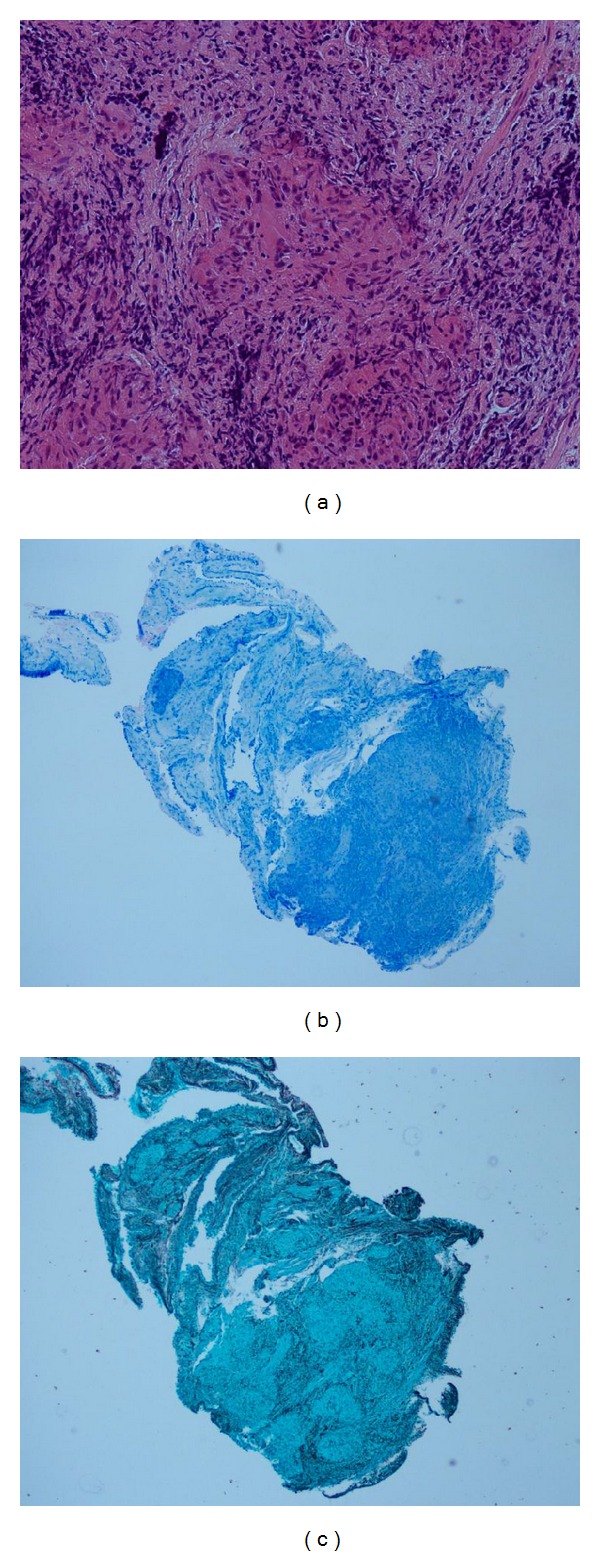
Histological analysis of transbronchial biopsies. (a) H&E stain showing presence of noncaseating granuloma with surrounding lymphocytes. (b) Ziehl-Nielson—no acid fast bacilli seen. (c) Grocott Stain—no fungal elements seen.

Histological analysis showed the presence of noncaseating granulomata with small number of surrounding lymphocytes. Special stains performed showed that there were no identifiable acid fast bacilli on Ziehl-Nielson stain or fungal element on PAS or Grocott stains (Figures 3(a)–3(c)). 

The investigations therefore make a diagnosis for miliary TB highly unlikely and thus suggestive of sarcoidosis given her clinical picture. Etanercept was discontinued and prednisolone commenced. 

## 2. Discussion

Tumour necrosis factor-*α* (TNF-*α*) is produced by a number of inflammatory cells such as macrophages and its role is implicated in the pathogenesis of granulomatous inflammation; blockade of TNF-*α* offers a potential role for targeted therapy. However, a series of nation-wide case reports have reported the association of sarcoid-like granulomatous disease after initiation of anti-TNF-*α* therapy, with disease reversal after discontinuation. A possible mechanism for this association is that anti-TNF-*α* therapies modulate a CD4+ Th1 cytokine response, key to the immunopathogenesis of sarcoidosis. CD4+ T cells interact with antigen presenting cells which initiate the formation and maintenance of granulomas, resulting in differentiation of selective Th1 cells secreting IFN-*γ* and IL-2 [1, 2]. In the chronic state, TNF-*α*, IL-12, IL-18 are the main cytokines produced. These cytokines are key in driving the Th1 commitment in the granulomatous process. Therefore blockade of TNF-*α* should have a therapeutic effect on sarcoidosis [1]. The immunopathogenesis remains poorly understood; a possible explanation is that there is overproduction of other cytokines that play a crucial role in granuloma formation with TNF-*α* blockade. Etanercept, a soluble TNF-*α* receptor fusion protein, is thought to enhance T-cell production of IFN-*γ* which is a key cytokine in the formation of granulomas in the acute stages of sarcoidosis [1, 3].

A number of studies have been carried out assessing the role of anti TNF-*α* in treatment of sarcoidosis, but their role remains questionable. A study using etanercept for stage II or III pulmonary sarcoidosis in seventeen patients was terminated early due to treatment failure when compared to conventional corticosteroid therapy [4]. Likewise, a double-blind randomised controlled study in eighteen patients with methotrexate resistant, corticosteroid dependent ongoing ocular sarcoidosis, showed a lack of steroid sparing effect and failure of ophthalmology global improvement [5]. These two studies using etanercept have failed to show treatment benefit in patients with progressive or methotrexate resistant sarcoidosis. 

Our patient continues to improve on a reducing regimen of oral corticosteroid therapy, with disappearance of symptoms and resolution of pulmonary nodulosis. However, the question remains on what to do next when inflammatory arthritis becomes active. There is limited data available regarding treatment options in such patients; with what we know is rechallenging with another anti TNF-*α*, the right thing to do? 

In contrast, two retrospective series reported symptomatic improvement in patients receiving infliximab when used in patients with chronic extrapulmonary disease (lupus pernio, uveitis, neurosarcoidosis), refractory to oral corticosteroid therapy or in patients who had not responded to etanercept [6]. However, a smaller study showed no difference in primary endpoints when used in patients with biopsy proven stage II-IV pulmonary sarcoidosis that had a suboptimal response or intolerance to oral corticosteroid therapy (minimum of 3 months treatment) [4]. In spite of this, a number of case reports to date are available highlighting the unexpected development of sarcoidosis following treatment with anti TNF-*α*, thus their role is controversial in granulomatous conditions. Consequently the use of corticosteroids is still the cornerstone of treatment in patients requiring systemic therapy.

Again, although the evidence is limited, leflunomide has been reported as an efficacious alternative therapy in treatment of sarcoidosis, thus offering the possibility of use for patients where sarcoidosis has developed following anti TNF-*α* therapy. Unfortunately our patient's psoriatic arthropathy failed treatment with leflunomide.

## 3. Conclusion

The evidence available remains limited in order to draw firm conclusions. Further studies are required to clarify the role of a second anti TNF-*α*, and until then corticosteroids remain the preferred option.
